# Vitamin D, Skeletal Muscle Function and Athletic Performance in Athletes—A Narrative Review

**DOI:** 10.3390/nu11081800

**Published:** 2019-08-04

**Authors:** Anna Książek, Aleksandra Zagrodna, Małgorzata Słowińska-Lisowska

**Affiliations:** Department of the Biological and Motor Basis of Sport, University School of Physical Education, Wrocław 51612, Poland

**Keywords:** 25(OH)D, calcidiol, calcitriol, muscle performance, muscle strength, physical activity, athlete

## Abstract

The active form of vitamin D (calcitriol) exerts its biological effects by binding to nuclear vitamin D receptors (VDRs), which are found in most human extraskeletal cells, including skeletal muscles. Vitamin D deficiency may cause deficits in strength, and lead to fatty degeneration of type II muscle fibers, which has been found to negatively correlate with physical performance. Vitamin D supplementation has been shown to improve vitamin D status and can positively affect skeletal muscles. The purpose of this study is to summarize the current evidence of the relationship between vitamin D, skeletal muscle function and physical performance in athletes. Additionally, we will discuss the effect of vitamin D supplementation on athletic performance in players. Further studies are necessary to fully characterize the underlying mechanisms of calcitriol action in the human skeletal muscle tissue, and to understand how these actions impact the athletic performance in athletes.

## 1. Introduction

Recent years have seen an increased interest in the research studies investigating vitamin D status in athletes. The growing number of scientific reports suggests a pleiotropic nature of vitamin D, suggested by the demonstration of vitamin D receptors (VDRs) nearly in every nucleated cell of our bodies. After binding with the membrane and nuclear VDRs, calcitriol may play a number of significant functions in the body, including effects on bone mineralization, normal function of the nervous, immune, endocrine and cardiovascular systems, as well as hormone production [1,2], regulation of the expression of over 900 gene variants [3] and on the normal function of the muscular system [1].

Calcitriol affects osteoblast function through various mechanisms, for example regulation of phosphate homeostasis by increasing the synthesis of fibroblast growth factor 23 (FGF23), and stimulation of mitogen-active protein kinase signaling, which can improve mechanical load response. Current evidence documents that bone cells can produce 1,25-dihydroxyvitamin D from the 25(OH)D precursor and that this activity may be responsible for the skeletal effects of circulating 25(OH)D. It is well known that athletes have a higher bone mineral density (BMD) compared to people leading a sedentary lifestyle. Any increase in body weight caused by training contributes to the process of bone remodeling and mechanically creates an appropriate bone structure. It is proposed to stimulate musculoskeletal loading through dynamic, high-intensity physical activity to compensate for low levels of 25(OH)D, with poor bone health in athletes. However, unencumbered athletes are susceptible to the same harmful skeletal effects and are more exposed to low BMD when vitamin D deficiency is found [1].

Vitamin D also affects both innate and adaptive immunity. VDRs are found in most cells of the immune system, including Treg cells, neutrophils, dendritic cells, B cells and macrophages [4]. The activation of the immune system can be regulated by circulating 25(OH)D and induced by activation of the toll-like receptor cascade in the presence of pathogenic bacterial microbiota. Vitamin D has been found to increase gene expression for broad-spectrum antimicrobial peptides (AMPs), which are important regulators of innate immunity. Calcitriol contributes to the immunomodulatory effect on T and B cells in acquired immunity. AMPs are crucial proteins for innate immunity and help defend against acute infections, including influenza and the common cold [1,5]. Differences in vitamin D concentrations may potentially affect the immune response. Studies have shown negative associations between vitamin D levels and the incidence of upper respiratory tract infections (URTI) in athletes especially during high-intensity exercise or prolonged and strenuous training periods [6,7].

Calcitriol activates many metabolic processes in the muscular tissue, exerting its actions via two types of receptors. This results in the stimulation of protein synthesis and an increased number of type II muscle cells, both of which lead to the increased muscle contraction velocity and strength. The combined effect of the activated proteins leads to muscle contraction. Calcitriol also plays an additional role in the proliferation and differentiation of muscle cells, and in the inhibition of their apoptosis [2,4,8]. This may be associated, in a multidirectional and multifactorial manner, with physical performance of athletes. Vitamin D deficiency may contribute to myopathy, decreased muscle tone, and imbalance and degradation of type II muscle fibers. This may have a negative impact on muscle strength, power and work [9,10].

The available literature includes a large number of reports on the assessment of vitamin D status in athletes. Many studies, particularly those conducted during the winter season, showed serum 25(OH)D levels that were below the recommended range [11]. In line with the vitamin D supplementation guidelines, in order to achieve the pleiotropic effects of vitamin D, 25(OH)D values should be maintained at the target level of 30 ng/mL (75 nmol/L) [12]. The appropriate choice of the supplementation dose, which depends on the individual’s health status (body weight, age, previous and present illnesses, and ethnicity) allows to reach an optimal serum 25(OH)D concentration and may have beneficial effects on health and performance-related variables.

The aim of this paper is to present the latest evidence on the relationship between vitamin D levels, skeletal muscle function and athletic performance in athletes. Furthermore, we provide evidence on the effect of vitamin D supplementation on physical performance in players.

## 2. Vitamin D and Skeletal Muscle Function

The role of vitamin D in the functioning of the muscle tissue is associated with the large number of VDRs found there [13]. The studies on human skeletal muscles show the presence of VDR in this tissue and confirmed that expression of VDR is crucial for effective uptake of calcitriol by muscle cells [14]. 1α25(OH)_2_D_3_ may exert its effects on muscles directly and indirectly, via two types of receptors: The nuclear and the membrane receptors. Calcitriol binds to VDR, in what contributes to the conformational changes that allow VDR to interact with a heterodimeric partner, retinoid X receptor (RXR). The 1,25D-VDR-RXR complex is translocated to the nucleus and binds to vitamin D response elements (VDRE), which finally effects in activation of gene transcription [13,14]. Upon binding with the nuclear receptor, calcitriol affects the cell directly, which results in changes in the gene transcription of mRNA and subsequent de novo protein synthesis. At the nuclear level, the activation of VDR induces the heterodimerization between the active VDR and an orphan steroid receptor known as RXR. The formation of this heterodimer facilitates the interaction between the receptor’s zinc finger region with DNA activating the protein transcription process [13,14]. This genomic pathway has been observed to influence the proliferation and differentiation of skeletal muscles and in the inhibition of apoptosis [13,15,16]. A trigger for these phenomena is the acceleration of specific kinases via stimulation of mitogen-activated protein kinase (MAPK). This may result in the proliferation of muscle cells and muscle growth [17]. 1,25(OH)_2_D affects the myogenic differentiation protein MYOD1 and myogenic regulatory factors. MYOD1 controls the process of muscle cell differentiation and plays a key role in muscle fiber regeneration by means of increasing their diameter [18].

Upon binding with its membrane receptor, calcitriol affects the cell indirectly, activates several interacting second-messenger pathways, resulting in cellular effects within seconds to minutes confirming 1,25(OH)_2_D’s role in modulating muscle contractility [17,19]. This non-genomic pathway has been found to results in an influx of calcium ions into the cell, regulation of the intra- and extracellular levels of this ion, homoeostasis of phosphorus-containing compounds and the stimulation of parathydroid hormone (PTH) secretion. The authors suggest that this could increase muscle force, strength and contraction rate [15,17].

In addition, vitamin D decreases the expression of myostatin, while stimulating the formation of follistatin and insulin-like growth factor-2 [2,8,19,20].

Vitamin D may also affect the diameter and number of type II muscle fibers. Vitamin D deficiency has been shown to be able to lead to decrease the concentration of intramyonuclear VDR, VDR gene expression level and contribute to a myopathy caused by type IIA muscle fiber atrophy. Calcitriol stimulates the synthesis of specific muscle proteins, which may result in increased muscle mass and strength. Hypertrophy of type IIB muscle fibers may affect exercise capacity of athletes. It is also noteworthy that type II fibers generate a faster muscle contraction and a greater force compared to type I muscle fibers [14,21]. Hence the ability to perform short high-power exercises such as sprints, jumps, rapid changes of movement, direction or stopping, is closely associated with type II fibers. Vitamin D deficiency may contribute to type II muscle fiber atrophy. This phenomenon is mainly observed in the elderly [4,10,22,23].

## 3. Vitamin D, Athletic Performance and Maximal Oxygen Uptake in Athletes

In the available literature over the past few years, there have been reports on the association of vitamin D with muscle strength and exercise performance in athletes. The results of these studies are inconclusive [1,24,25,26,27]. Table 1 lists the characteristics of the included studies.

No relationship has also been demonstrated between 25(OH)D levels on the one hand and hand grip strength and muscle strength measured under isokinetic conditions, nor vertical jump height in female DanceSport competitors, female gymnasts or female swimmers [36]. This is in contrast to a study conducted by another team, where a statistically significant positive correlation was documented between calcidiol levels and hand grip strength in professional judo athletes. The authors of this study emphasized that the unique features of judo require a considerable hand grip strength, which could point to a relationship between vitamin D levels and strength of the muscle group under investigation [34].

VDRs have also been demonstrated in the myocardium and cardiac conduction tissue [37]. This shows that calcitriol may be associated with maximal oxygen uptake (VO_2_max) through oxygen transport ability and oxygen utilization in various tissues [38]. Generally, a positive correlation has been observed between 25(OH)D levels and VO_2_max in physically inactive individuals [39,40,41], and studies in professional athletes have been inconclusive. Koundourakis et al. [32] has shown an association of 25(OH)D levels with VO_2_max in 67 elite football players. Forney et al. [30] studied a group of physically active students and observed that those with vitamin D levels of > 35 ng/mL had significantly higher VO_2_max levels than those with lower 25(OH)D levels. It is notable that this association was only observed in males. Fitzgerald et al. [29] observed no association of 25(OH)D with VO_2_max in 52 professional ice hockey players. It should be emphasized that the relationship between vitamin D levels and VO_2_max is reversely correlated with increased levels of physical activity and fitness level [40].

This may suggest that the relationship between vitamin D and maximal oxygen uptake is more pronounced in individuals who pursue sports for leisure compared to elite professional athletes. The mechanism underlying the VO_2_max increase by calcitriol is unclear. This may be related to the activation of cytochrome P450 (CYP) activation by 1,25-dihydroxy vitamin D. CYP contains heme as the prosthetic group, which may potentially increase hemoglobin’s binding affinity for oxygen [42].

## 4. Effects of Vitamin D Supplementation on Athletic Performance in Athletes

The available literature shows that vitamin D supplementation may increase the number of VDRs in muscles, improve muscle performance and lower the risk of bone fracture, although these results have been documented mainly in the elderly [43,44,45]. The results of studies conducted in athletes are inconclusive. Some of them showed positive effects of vitamin D supplementation on muscle strength and exercise abilities in athletes [46,47], while others did not [48,49,50]. A detailed list of studies investigating vitamin D supplementation and athletic performance in athletes is presented in Table 2.

A beneficial effect has been demonstrated for an 8-week vitamin D supplementation at a dose of 5000 IU/day on 10 m sprint time and on vertical jump height compared to the placebo group [46]. Similar results have been observed by Wyon et al. [47], who demonstrated that a 4-month vitamin D supplementation increased isometric force and vertical jump height in female DanceSport competitors.

It is now recognized that calcitriol affects muscle function through the regulation of muscle protein synthesis, cell differentiation and cell proliferation, as well as the transport of calcium and phosphate across muscle cell membranes, while modulating phospholipid metabolism. Moreover, vitamin D may regulate mitochondrial function, dynamics and enzyme function in muscle cells [58,59]. Data shows that vitamin D deficiency negatively affects muscle function and contributes to proximal muscle weakness with a reduction in type II muscle fibers [10,13]. Vitamin D supplementation increases muscle fiber size, VDR percent in type II fibers, and the intramyonuclear VDR concentration [44]. Moreover, calcifediol supplementation may have positive effects on muscle strength by increasing mitochondrial function and inhibiting muscle atrophy [60,61]; a statistically significant improvement has been observed in upper and lower limbs, but these results have been obtained in elderly women [60]. This data suggests that a population with low baseline levels of vitamin D or the elderly might benefit more from vitamin D supplementation in terms of its effects on muscle function.

No effect of vitamin D supplementation on muscle power has been shown in the NASCAR car race team [52], nor athletes representing various sports disciplines (football, tennis, lacrosse, wrestling, baseball) [49] and professional rugby and football players [48]. Recent research has shown that there are no observable differences between supplemented and placebo groups under isokinetic conditions in athletes representing various disciplines [36,49], professional football players [56], and competitive swimmers [53]. In other studies of vitamin D, supplementation had no effects on sprint times. Speed abilities did not differ between supplementation and placebo groups for any of the outcome measures [46,50,54].

The mechanisms for the differential effect of vitamin D supplementation on lower and upper limb muscle strength are unclear. It is possible that VDR expression in various muscle groups can contribute to the differential effects between the lower and upper limb muscles [59,62]. Perhaps the methods used to assess the strength of upper limb muscles do not give sufficiently precise results, and thus do not record slight changes in the strength gain in the upper limbs. Moreover, the different methods/assays used to measure vitamin D status may have led to the different serum 25(OH)D values. Based on current evidence, vitamin D supplementation may have a positive effect on lower limb muscle strength in athletes, but no effects have been documented on upper limb muscle force, muscle power and sprint abilities. This might suggest that supplementation of vitamin D differently affects different muscle abilities and muscle groups in athletes.

## 5. Vitamin D Insufficiency and Deficiency in Athletes

In line with the latest guidelines, the normal ranges for serum 25(OH)D levels are defined as 30–50 ng/mL (75–125 nmol/L) or 40–60 ng/mL (100–150 nmol/L). Vitamin D insufficiency is defined as serum levels of 20–30 ng/mL (50–75 nmol/L) and vitamin D deficiency as serum levels below 20 ng/mL (<50 nmol/L) [12]. Lanteri et al. [63] has documented a high prevalence of vitamin D insufficiency and deficiency in athletes. Factors which may inhibit the synthesis of vitamin D in athletes include geographic location, skin pigmentation, indoor training, early- or late-day training and extensive sunscreen use.

A meta-analysis of the literature from 2008 to 2014 evaluated publications reporting a total of 2313 professional athletes. It showed that 56% of the athletes had an inadequate 25(OH)D concentration and that these were significantly lower during the winter period. The studies in question assessed athletes inhabiting areas of high and low levels of insolation. Vitamin D deficits were shown to be common in countries of low levels of insolation. It should, however, be noted that vitamin D deficiency is also found in countries of high levels of insolation, with the risk of insufficient 25(OH)D levels being much higher in the winter and spring periods compared to the autumn and summer periods [11,64]. It should be emphasized that vitamin D synthesis as a result of exposure to sunlight is considerably limited from October to April in areas between 42.2° to 52° N [38].

Athletes training indoor have been shown to have low 25(OH)D levels [6,34]. In general, individuals participating in indoor sports are at a higher risk of vitamin D insufficiency and deficiency, although studies have shown that both groups of athletes had reduced levels of vitamin D [11,65,66]. Lombardi et al. [67] has demonstrated that 32.9% of Italian professional soccer players had insufficient levels of vitamin D and 9% showed vitamin D deficiency. Krzywański et al. [68] studied 409 Polish athletes and found that 80% of those who trained outdoors and 84% of those training indoors had 25(OH)D deficits. Vitale et al. [69] also documented insufficient levels of calcidiol in 50.7% elite professional skiers with 29.6% showing deficient levels.

In cases of vitamin D deficiency, it is important to take into consideration the bioavailability of this vitamin and thus its binding to vitamin D-binding protein (VDBP). VDBP is the main vitamin D carrier. It binds a total of 85–90% of 25(OH)D and 1,25-dihydroxyvitamin D_3_ (the biologically active form of vitamin D) present in the circulation, and the remaining unbound fraction of 25(OH)D is considered bioavailable. Approximately 10–15% of the total quantity of 25(OH)D is bound with albumin, unlike the free amount of 25(OH)D, which is made up of 1% of the total amount vitamin D present in the circulation [70]. As the affinity of albumin for 25(OH)D or 1,25(OH)_2_D_3_ is lower than that of VDBP, the bioavailable 25(OH)D is made up of the loosely bound and the free fractions. In humans with a mutated VDBP gene, there may be a considerable interpersonal variation in the amount of bioavailable vitamin D [71]. As a result, differences in the signs and symptoms of vitamin D deficiency may be related to the genetic variation in VDBP [1,72,73]. The VDBP gene is highly polymorphic in various racial groups [1,14,74]. In order to increase the precision and accuracy of vitamin D measurements, we simply need to measure the free fraction of vitamin D, as this measurement is independent of these confounding factors and is therefore much better correlated with pathological conditions [75]. These findings may suggest that vitamin D status is better assessed by measuring free vitamin D and/or its bioavailable 25(OH)D metabolites.

## 6. Conclusions

Further research is necessary to characterize the true vitamin D status by simply measuring free vitamin D rather than total 25(OH)D. Importantly, it may be better to assess the relationship between free vitamin D, skeletal muscle function and exercise ability, to understand how these actions affect athletic performance.

## Figures and Tables

**Table 1:** Vitamin D, athletic performance and VO_2_max in athletes.

Authors	Study Population	Vitamin D Levels Mean ± SD [ng/mL]	Findings
**Dubnov-Raz et al. [28]**	*n* = 80 F + M, professional swimmers	29.49 ± 5.2	No association of 25(OH)D with hand grip strength
**Fitzgerald et al. [29]**	*n* = 52 M, professional ice hockey players	35.7 ± 8.9	No association of 25(OH)D levels with the test parameters: VO_2_max, HR, total duration of exercise
**Forney et al. [30]**	*n* = 39 F + M, physically active students	36.73 ± 3.2—F 33.02 ± 2.1—M	No association of 25(OH)D levels with the maximal muscle strength Association of 25(OH)D with VO_2_max (*p* = 0.018)
**Hamilton et al. [31]**	*n* = 342 M, professional football players	21.6 ± 4.3	Athletes with 25(OH)D levels of >30 ng/mL had higher values of peak torque in the non-dominant leg compared to those with 25(OH)D levels of ≤10 ng/mL (*p* = 0.015),
**Koundourakis et al. [32]**	*n* = 67 M, professional football players	34.4 ± 7.08 after 6 weeks 47.21 ± 13.5	Association of 25(OH)D levels with vertical jump (SJ (*p* < 0.001), CMJ (*p* < 0.001)), 10 m (*p* < 0.001), 20 m sprint times (*p* < 0.001), and VO_2_max (*p* = 0.006)
**Książek et al. [33]**	*n* = 43 M professional football players	16.9 ± 8.4	Athletes with 25(OH)D levels of >20 ng/mL had higher values of peak torque compared to those with 25(OH)D levels of ≤20 ng/mL (*p* ≤ 0.05) A statistically significant positive correlation has been shown between 25(OH)D levels and peak torque in the knee joint during extension of the left lower limb (at an angular velocity of 150°/s) (*p* ≤ 0.05)
**Książek et al. [34]**	*n* = 25 M, elite judo athletes	17.4 ± 5.2	Association of 25(OH)D with hand grip strength (*p* ≤ 0.05), power (SJ) (*p* ≤ 0.05) and total work (*p* ≤ 0.05) measured under isokinetic conditions
**Zeitler et al. [35]**	*n* = 284 F, 297 M, healthy recreational athletes	27.2 ± 10.9—F 24.8 ± 10.2—M	M with 25(OH)D levels <20 ng/mL had significantly lower submaximal physical performance measured on a treadmill ergometer than those with normal levels (*p* = 0.045) Association of 25(OH)D levels with maximal (*p* = 0.003) and submaximal physical performance (*p* = 0.002) in M In F no significant differences in maximal and submaximal physical performance were detected

F: female; M: male; VO_2_max: maximal oxygen uptake; HR: heart rate; SJ: squat jump; CMJ: counter movement jump.

**Table 2:** Effects of vitamin D supplementation on athletic performance and VO_2_max in athletes.

Authors	Study Population	Dose of Supplemented Vitamin D	Findings
**Close et al. [48]**	*n* = 30 M, professional rugby and football players	20,000 or 40,000 IU/week vs. placebo for 6 or 12 weeks	No association of 25(OH)D with muscle strength and power
**Close et al. [46]**	*n* = 61 M, professional football, rugby players, horse racing	5000 IU/day vs. placebo for 8 weeks	Increased vertical jump height (*p* = 0.008) and decreased 10 m sprint time (*p* = 0.008) in subjects receiving vitamin D supplementation versus placebo
**Mitchell [36]**	*n* = 54 F, DanceSport competitors, professional gymnasts and swimmers	50,000 IU/month vs. placebo for 6 weeks	No association of 25(OH)D with hand grip strength, muscle strength measured under isokinetic conditions and vertical jump
**Jastrzębski [51]**	*n* = 14 M, professional rowers	6000 IU/day vs. placebo for 8 weeks	Increased VO_2_max levels (*p* < 0.05) in athletes receiving vitamin D supplementation compared to the placebo group
**Nieman et al. [52]**	*n* = 28 M, NASCAR car race team	3800 IU/day vs. placebo for 6 weeks	No association of 25(OH)D levels with muscle strength measured under isokinetic conditions, vertical jump, maximal force and power measured in the Wingate test
**Shanely et al. [49]**	*n* = 33 M, professional football, tennis, lacrosse and baseball players and professional wrestlers	600 IU/day vs. placebo for 6 weeks	No association of 25(OH)D with muscle strength measured under isokinetic conditions or vertical jump
**Dubnov-Raz et al. [53]**	*n* = M, adolescent competitive swimmers	2000 IU/day vs. placebo for 12 weeks	No differences of handgrip strength, swimming performance at several speeds between placebo and supplemented group
**Wyon et al. [47]**	*n* = 24 F, professional ballet dancers	2000 IU/day vs. placebo for 4 months	Increased isometric force (*p* < 0.01) and vertical jump height (*p* < 0.01) in athletes receiving vitamin D supplementation compared to the placebo group
**Jastrzębska et al. [54]**	*n* = 36 M, well-trained soccer players	5000 IU/day vs. placebo for 8 weeks	No differences of peak power, total work capacity, 5, 10, 20, 30 m sprint running times, SJ, and CMJ between placebo and supplemented group
**Jastrzębska et al. [55]**	*n* = 36 M, well-trained soccer players	5000 IU/day vs. placebo for 8 weeks	The supplemented group demonstrated a significant increase in VO_2_max compare to placebo group (*p* < 0.0001)
**Todd et al. [56]**	*n* = 43 M, football players	3000 IU/day vs. placebo for 12 weeks	No association between 25(OH)D and left/right hand grip strength, CMJ height
**Wyon et al. [45]**	*n* = 22 M, judo athletes	150,000 IU once vs. placebo for 8 days	The treatment group demonstrated a significant increase in muscle strength between days 1 and 8 (*p* = 0.01)
**Fairbairn et al. [50]**	*n* = 57 M, professional rugby union players	50,000 IU once a fortnight vs. placebo for 11–12 weeks	No differences of 30 m sprint time between placebo and supplemented group. The treatment group demonstrated a significant increase in weighted reverse-grip Chin-up 1RM (*p* = 0.002). No association between 25(OH)D, bench pull 1RM and bench press 1RM
**Skalska et al. [57]**	*n* = 36 M, young soccer players	5000 IU/day vs. placebo for 8 weeks	No differences of physical activity indicators in the supplemented and un-supplemented groups

F: female; M: male; VO_2_max: maximal oxygen uptake; SJ: squat jump; CMJ: counter movement jump; 1RM: one repetition maximum.
